# Ethylene: A Master Regulator of Plant–Microbe Interactions under Abiotic Stresses

**DOI:** 10.3390/cells12010031

**Published:** 2022-12-21

**Authors:** Kirti Shekhawat, Katja Fröhlich, Gabriel X. García-Ramírez, Marilia A. Trapp, Heribert Hirt

**Affiliations:** DARWIN21, Center for Desert Agriculture, Biological and Environmental, Science and Engineering Division, King Abdullah University of Science and Technology (KAUST), Thuwal 23955-6900, Saudi Arabia

**Keywords:** Ethylene, plant-microbe interactions, abiotic stresses, transcriptome, reactive oxygen species

## Abstract

The plant phytohormone ethylene regulates numerous physiological processes and contributes to plant–microbe interactions. Plants induce ethylene production to ward off pathogens after recognition of conserved microbe-associated molecular patterns (MAMPs). However, plant immune responses against pathogens are essentially not different from those triggered by neutral and beneficial microbes. Recent studies indicate that ethylene is an important factor for beneficial plant–microbial association under abiotic stress such as salt and heat stress. The association of beneficial microbes with plants under abiotic stresses modulates ethylene levels which control the expression of ethylene-responsive genes (*ERF*), and *ERF*s further regulate the plant transcriptome, epi-transcriptome, Na^+^/K^+^ homeostasis and antioxidant defense mechanisms against reactive oxygen species (ROS). Understanding ethylene-dependent plant–microbe interactions is crucial for the development of new strategies aimed at enhancing plant tolerance to harsh environmental conditions. In this review, we underline the importance of ethylene in beneficial plant–microbe interaction under abiotic stresses.

## 1. Introduction

Plant–microbe interactions are an important part of our living ecosystem as they maintain environmental sustainability. Plants harbor a complex variety of microorganisms ranging from “mutualists” to “pathogens”. The symbiotic and mutualistic interactions with beneficial microbes show positive communication whereas the interactions with pathogenic microbes represent a negative one [1,2]. In the process of any plant–microbe interaction, plants produce several compounds that influence these interactions. Phytohormones regulate many processes in plants. Among these, ethylene, a gaseous hormone, plays a central part in pathogenic as well as beneficial plant–microbe interactions [3,4,5,6]. In plants, the production of ethylene is subject to several biotic and abiotic factors that affect many physiological and developmental processes, indicating its role in plant adaptation to environmental changes [3,7,8]. Ethylene biosynthesis starts with the conversion of methionine into S-adenosyl-methionine (SAM) by SAM synthetases. SAM further gets converted into the ethylene precursor 1-aminocyclopropane-1-carboxylic acid (ACC) via ACC synthases, and finally, ACC is converted into ethylene by ACC oxidases. Ethylene then diffuses out of and into plant cells [9,10,11]. The perception of ethylene signaling takes place at the endoplasmic reticulum membrane where it initiates a signaling cascade that results in ETHYLENE RESPONSE FACTORs (ERFs)-mediated transcriptional regulation of ethylene-responsive genes in the nucleus. In *Arabidopsis*, the ethylene receptors ETHYLENE RESPONSE SENSOR1 (ERS1), ERS2, ETHYLENE RESPONSE1 (ETR1), ETR2, and ETHYLENE INSENSITIVE4 (EIN4) act as negative regulators of the ethylene signaling pathway. In the absence of ethylene signaling, these ethylene receptors activate CONSTITUTIVE TRIPLE RESPONSE1 (CTR1), which inhibits EIN2, a positive regulator of ethylene signaling, by phosphorylating the C-terminus of EIN2. In contrast, the presence of ethylene inactivates the ethylene receptors and therefore inhibits the activation of CTR1. Consequently, dephosphorylated and cleaved EIN2 C-terminus (CEND) enters the nucleus where it promotes the activity of transcriptional master-regulators of ethylene signaling, ethylene-insensitive3/ethylene-insensitive3-like1 (EIN3/EIL1) which controls the transcription of ethylene-responsive target genes, such as ETHYLENE RESPONSE FACTORs. ERFs are AP2domain-containing transcription factors (TFs) that regulate several processes via regulating the expression of several genes of the stress response, development, growth and hormone-related mechanisms [12,13,14]. For instance, in *Arabidopsis*, ethylene regulates auxin distribution which regulates ROS and ROS further has an impact on the epigenetic mechanisms of gene regulation [15,16]. Ethylene also shows cell-type-specific responses. A study by Vaseva et al. used a targeted expression approach to map the site of ethylene response by a number of cell-type-specific promotors to the F-box proteins EBF1 and 2. In phenotypic experiments, the epidermis of both roots and shoots has been revealed as the main site of ethylene response which also impacts the cortical layer mainly, via crosstalk with auxin [17]. Ethylene is also reported to inhibit the proliferation of the *Arabidopsis* root meristem cells [18]. Comparing the transcriptome of different leaf tissues revealed that ethylene/auxin crosstalk plays a role in mitochondrial regulation mainly in epidermis cells [19]. Ethylene not only regulates several aspects of plant growth but also participates in several plant–microbe interactions, thereby impacting microbial assembly [4,20,21]. In this context, one of the very first events during plant–pathogen interactions is a rapid increase in ethylene biosynthesis in an attempt to ward off the pathogen. However, plants also involve ethylene in beneficial plant–microbe interactions. Some helpful microbes need ethylene to provide plant growth promotion under abiotic stresses; in contrast, other microbes are known to produce ACC deaminase which might reduce ethylene levels to provide a beneficial effect. This suggests a concentration-dependent effect of ethylene on plant growth during plant-microbial interactions and also indicates the possibility that ethylene may play a subtle negative role in microbe-mediated stress tolerance in plants [22,23,24,25]. Therefore, a more refined discussion is necessary to understand the actual involvement of ethylene during plant–microbe interactions. In this article, we first review the importance of ethylene for plant stress tolerance and then provide an overview of how ethylene is involved in plant–microbial interactions, thereby shifting the plant response toward stress adaption.

## 2. Ethylene and Plant Abiotic Stresses

Being sessile organisms, plants can face survival threats from environmental perturbations. However, under such circumstances, plants still try to adjust their lifestyle by developing a series of strategies resulting in a stress-specific phenotype [26,27]. This integration of the phenotypic response to the environmental status is coordinated with help of phytohormone signaling. In response to abiotic stresses, plants produce several phytohormones including ethylene, which is crucial for plant growth and development under different abiotic stresses including salt, hypoxia and heat stress (HS).

### 2.1. Salt Stress

**Figure 1:**
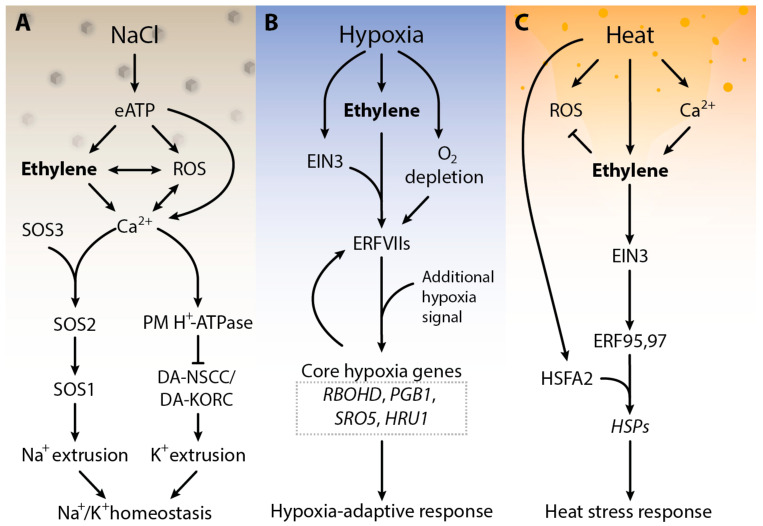
Schematic presentation of salt (**A**), hypoxic stress (**B**) and heat stress (HS) (**C**) signaling response in plants. Under salt stress, when Na^+^ ions are sensed, extracellular ATP is generated, which results in the accumulation of ROS and cytosolic calcium (Ca^2+^)cyt. Consequently, the extracellular eATP triggers ethylene signaling which interacts with ROS and (Ca^2+^)cyt, ROS and ethylene, and ethylene and (Ca^2+^)cyt regulates the plasma membrane Na^+^/H^+^ antiporter and H^+^-ATPase. Additionally, ethylene can also directly initiate the SOS signaling pathway. Subsequently, the plasma membrane Na^+^/H^+^ antiporter helps to eliminate the excess Na^+^ and H^+^-ATPase prevents DA-KORCs/DA-NSCCs to control cytosolic K^+^ leakage, by balancing K^+^/Na^+^ homeostasis in salt stress. Upon submergence, plants accumulate ethylene within minutes due to restricted gas diffusion. Under hypoxia (**B**), the lack of oxygen stabilizes the ERF-VII proteins. The stable ERF-VII proteins activate the transcription of hypoxia-responsive genes in the nucleus. Under heat stress (**C**), HS changes plasma membrane integrity which results in calcium influx. Heat stress and Ca^2+^ influx activate ethylene signaling, which activates ERF95,97 via EIN3. ERFs bind to the promoter regions of *HSP* genes to activate their transcription and therefore results in enhanced thermotolerance. Abbreviation—ROS: reactive oxygen species, (Ca^2+^)cyt: cytosolic calcium, PM: plasma membrane, SOS: salt over sensitive, DA-KORCs: depolarization-activated K^+^ outward rectifying channels; DA-NSCCs: depolarization-activated non-selective cation channels. RBOHD: respiratory burst oxidase protein D, PGB1: Phytoglobin 1, *SRO5*: Similar to RCD one 5, HRU1: Hypoxia responsive universal stress protein 1. HS: heat stress, ERF: ethylene response factors, EIN: ethylene insensitive, HSE: heat shock element, HSP: heat shock protein regulate the HRS. Arrows indicate positive regulation (heat stress transcription factor 1).

Salt stress is one of the main dangers to crop plant productivity worldwide [28,29]. Plants manage salt stress by using the SOS (salt over sensitivity) signaling pathway. Salinity stress provokes the buildup of Ca^2+^, which is recognized by SOS3, the calcium-bound SOS3, then further activates the SOS2 protein kinase. The activation of SOS2 results in the phosphorylation of SOS1, a plasma membrane (PM) Na^+^/H^+^ antiporter; SOS1 then transports Na^+^ out of the cytosol (Figure 1A). Experimental evidence indicates the key role of ethylene as a regulator of plant salt stress. In *Arabidopsis*, salt stress suppresses the expression of *ETR1* expression. In addition, *etr1* loss-of-function mutants show improved salt tolerance and germinate earlier than wild type (WT) plants. In contrast, *etr1* gain-of-function mutants show more sensitivity to salt stress, suggesting that this gaseous plant hormone works as the main modulator of salt stress response in plants [30,31,32]. An overproduction of endogenous ethylene or exogenous treatment of the ethylene precursor ACC increases salinity stress tolerance in various plants including *Arabidopsis*, tomato, grapevine and maize [33,34,35]. Ethylene modulates salinity stress responses by Na^+^/K^+^ homeostasis. Homeostasis of Na^+^ ions maintains membrane integrity, plant water content and photosynthesis in plants [36,37]. The maintenance of the Na^+^/K^+^ homeostasis by ethylene involves intricate signaling between ethylene, H_2_O_2_, and cytosolic calcium (Ca^2+^cyt) and extracellular ATP (eATP). When *Arabidopsis* roots sense the increased concentration of sodium ions, eATP is produced, resulting in the accumulation of ROS in apoplast, chloroplasts, mitochondria and peroxisomes. In the meantime, eATP triggers the activation of ethylene signaling via the upregulation of *EIN3*. Activation of ethylene signaling interacts with ROS and Ca^2+^cyt to regulate the PM Na^+^/H^+^ antiport SOS systems. Ethylene can also directly activate the SOS pathway by enhancing the transcription of SOS1/2/3. As a result, the PM Na^+^/H^+^ antiporter helps to omit the excess Na^+^, while H^+^-ATPase inhibits DA-KORCs/DA-NSCCs (depolarization-activated K^+^ outward rectifying channels/depolarization-activated non-selective cation channels) to limit cytosolic K^+^ leakage, overall maintaining Na^+^/K^+^ homeostasis under salinity stress (Figure 1A) [38]. Ethylene also helps to maintain stomatal conductance, water use efficiency and osmotic adjustment to protect the plants from salinity stress [39]. Research on the role of ethylene in plant salt stress tolerance suggests that fine-tuning of ethylene may be necessary for salt stress tolerance in plants as ethylene levels may positively or negatively affect plant responses to salt stress [40,41,42]. Although other studies have shown the role of other hormones in plant stress tolerance and indicate a cross-talk between ethylene with other phytohormones during salinity stress, a thorough mechanism is still unclear and needs further investigation.

### 2.2. Hypoxia

Hypoxia (insufficient oxygen availability) in plants usually arises as the result of heavy rains and subsequent flooding [38,39,43,44]. Because of limited gas diffusion underwater, submerged plants face a shortage of oxygen and therefore existence depends on molecular responses that increase plant hypoxia tolerance. In submerged plant tissues the restricted gas diffusion results in ethylene accumulation. This ethylene accumulation can occur before the onset of hypoxia, making it a suitable signal for submergence. In flooded plants, ethylene regulates adaptive responses to submergence by inducing morphological changes that help to survive in hypoxia. Ethylene was shown to accelerate and enhance hypoxia response genes through enhanced steadiness of specific ethylene response transcription factors (ERFs group VII) (Figure 1B) [43,45]. During low levels of oxygen, the expression of genes related to nitrogen, carbon glycolysis and anaerobic respiration increases in an ethylene-dependent manner. The mechanism behind ethylene-mediated hypoxia responses in plants includes the incorporation of ethylene signaling with the plant’s low oxygen-sensing machinery. Under normal oxygen levels, class VII ethylene response factors become degraded upon oxidation of the N-terminal amino acid cysteine by plant cysteine oxidases in the presence of O_2_ [46,47,48]. A decline in O_2_ levels stabilizes ERFVIIs and other cysteine-initiating proteins, leading to the expression of core hypoxia genes and hypoxia acclimation such as *RBOHD*, *PGB1*, *SRO5* and *HRU1* (Figure 1B) [46,49,50,51]. However, the increase in ERFVIIs alone does not induce the transcription of central hypoxia genes until an extra hypoxia signal is introduced, showing that ethylene alone cannot induce the expression of the core hypoxia genes under normal oxygen conditions [52,53,54]. In this context, as soon as O_2_ levels drop, the transcription of hypoxia genes is stronger and quicker compared to control plants without prior ethylene exposure. Taken together, these outcomes show that ethylene accumulation primes plant tissues for potentially impending hypoxia. However, how precisely plants integrate low O_2_ signaling and ethylene during submergence to enhance survival remains elusive.

### 2.3. Heat Stress

A rise in temperature above threshold levels causes cell damage by affecting physiological, cellular and molecular functions. High temperatures induce membrane defect, DNA damage, protein denaturation and reactive oxygen species (ROS) buildup which consequently results in oxidative stress, and hence, programmed cell death of the plant [55,56,57]. Under such conditions, plants deploy ethylene biosynthesis and signaling to survive. Ethylene is an important regulator of HS responses [58,59]. Genes related to ethylene signaling such as ERFs, bind to heat stress transcription factor-2 *(HSFA2)* and activate the downstream cascade of HS management. In this context, *ERF95* and *ERF97* have been shown to bind directly to the promoter region of *HSFA2*, which in turn modulates the expression of heat shock proteins (HSPs) to provide plant thermotolerance (Figure 1C) [60,61,62]. In addition, ethylene also regulates the metabolism of ROS via modulating osmo-protectants and the antioxidant defense system. Ethylene-mediated signaling was involved in the enhancement of thermotolerance in rice and *Arabidopsis* seedlings by decreasing oxidative damage and maintenance of chlorophyll content under HS. Rice plants treated with ethylene precursor showed reduced levels of cell membrane oxidation and ion leakage under heat treatment, conferring improved thermotolerance. Higher expression levels of heat shock transcription factors *HSFA1a* and *HSFA2a*, *c*, *d*, *e*, *f* and ethylene-signaling-related genes such as *EIN2* and *EIN3* were observed in rice seedlings treated with ethylene precursor under HS than in rice seedlings under mock HS treatments [60]. In all these examples it seems that ethylene signaling-mediated HS alleviation mainly involves *ERF*s. However, the mechanisms underlying the crosstalk between heat and ethylene signaling remain unknown.

## 3. Ethylene and Plant–Microbe Interactions

As mentioned before, ethylene is the main regulator of plant life in many aspects, including numerous mechanisms by which plants communicate with a pathogen or beneficial microbe. Ethylene regulates these plant-microbial interactions by regulating the expression of ethylene-responsive genes by ERFs or by interactions with other phytohormones [4,63,64]. Many studies have shown the importance of ethylene in the establishment of plant interactions with pathogens and beneficial microbes which we discuss below.

## 4. Ethylene and Pathogenic Plant–Microbe Interactions

Plants possess an innate immune system in which each cell can detect molecular patterns as danger signals by a multilayered interface of pattern recognition receptors (PRRs) at the cell surface, which in turn activate pattern-triggered immune response (PTI), defending the plant against non-host adapted pathogens [65]. PRRs can bind to microbially derived extracellular molecules, which are often highly conserved across whole classes of microbes (microbe-associated molecular patterns, MAMPs). Host-adapted pathogens can evade plant immune responses by secreting effectors into the apoplast of the plant. In an evolutionary arms race, plants developed effector-binding receptors (nucleotide-binding domain leucine-rich repeat-containing (NLR) protein receptors) which induce effector-triggered immunity (ETI). The most studied MAMP is bacterial flagellin (flg22), which is detected in *Arabidopsis* by the PRRs FLAGELLIN-SENSING 2 (FLS2). As a response to pathogens, the plant produces ethylene which inhibits the growth of certain pathogens by regulating the transcription of pathogen response genes. The treatment of plants with flg22 induces ethylene biosynthesis via MAP kinases 3 and 6-mediated phosphorylation of the rate-limiting ET biosynthetic enzymes *ACS2* and *ACS6.* These MPKs also phosphorylate EIN3 resulting in its stabilization. Ethylene activates various transcription factors such as *ERF1* and OCTADECANOID-RESPONSIVE ARABIDOPSIS *AP2/ERF 59* (*ORA59*) that are involved in the regulation of immunity-associated genes [66,67]. EIN3 and EIL1 are also involved in the positive feedback loop by binding the promoter region of *FLS2* [68]. Furthermore, plants disturbed in their ethylene signaling such as the *ein2* mutant are impaired in PTI, resulting in increased susceptibility towards virulent *Pseudomonas syringae*. Application of exogenous ethylene or constitutive expression of ERF1 safeguards the plants against the necrotrophic fungus *Botrytis cinerea*, in contrast, ethylene-insensitive mutants (e.g., *ein2*) display enhanced susceptibility to *B. cinerea* [69]. The detection of pathogens also affects the production of other phytohormones like salicylic acid (SA), abscisic acid (ABA) and jasmonic acid (JA). An orchestra of these phytohormones and other signaling pathways form a complex regulatory network to fine-tune specific defenses against distinct pathogens [70,71]. Interestingly, the role of these plant defense hormones is different in dicots and monocots [72,73]. In dicots, SA is involved in the defense against biotrophic pathogens such as *P. syringae*, while jasmonic acid and ethylene work together in defense against necrotrophic pathogens such as *Alternaria brassicae* [70,74]. In monocots, ethylene is effective against pathogens with diverse lifestyles. Ethylene also plays a modulating role in plant defense regulating other phytohormones [75,76,77]. JA and ethylene work synergistically. The ERFs have been reported to integrate signals from ethylene and JA [78]. Other typical marker genes expressed after ethylene and JA detection are *POTLX3* (lipoxygenase) [79], *ACS* (ethylene synthesis gene) [80], *THI2.1* (thionin) [81], *PDF1.2* (defensin); *PR-3* (chitinase); *PR-4* (hevein-like protein) [81,82], *PR-6* (proteinase inhibitor) [83], *PR-9* (peroxidase) [84]. SA and ethylene act antagonistically and their biosynthesis pathways can be mutually repressed. NONEXPRESSER OF PR GENES 1 (NPR1) a core component of SA signaling, directly interacts with EIN3, blocking the transcription of EIN3-induced genes [85]. In turn, EIN3 and EIL1 directly bind to the *SID2* promotor downregulating pathogen-induced biosynthesis of SA and enhancing disease susceptibility to *P. syringae* [86]. To understand this discrepancy, further analyses of the function of ET in crosstalk with other hormones are required including experiments to investigate whether ethylene interacts with other hormones in a specific temporal and spatial manner.

## 5. Ethylene and Beneficial Plant–Microbe Interactions

**Figure 2:**
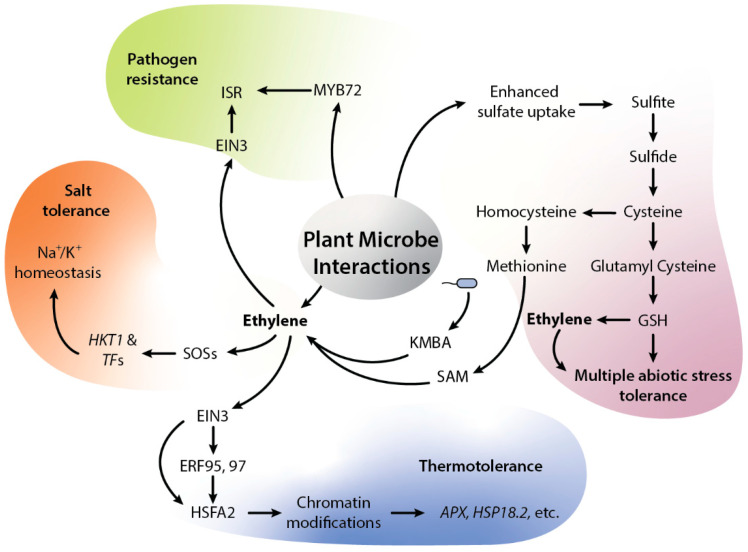
Diagrammatic representation of ethylene-mediated beneficial plant-microbial interactions. Microbes such as SA187 can trigger ethylene signaling via sulfur metabolism by uptake of sulfate, which further becomes converted into sulfite, sulfide, and cysteine. Further, cysteine can be used to generate either methionine or glutathione (GSH). GSH can be used in ROS scavenging, while methionine can regulate sulfur regulon via its conversion into SAM, ACC and ethylene. SA187 also produces KMBA, which plants can use to produce ethylene. In addition, KMBA contains sulfur which can be converted into SAM and SAM further modulate ethylene signaling. Ethylene further regulates salt stress via Na^+^/K^+^ homeostasis. Similarly, SA187-modulated ethylene can reprogram the plant transcriptome under HS. SA187-produced compounds activate ethylene signaling in *Arabidopsis,* which can enhance the plant thermotolerance via increased ERF-mediated expression of *HSFA2* and *HSPs* genes. Fascinatingly, the presence of microbes can induce the expression of *APX2* and *HSP18.2* memory genes via EIN2- and HSFA2-dependent H3K4me3 modification. The beneficial microbes can also induce systemic resistance (ISR) via ethylene. Ethylene and MYB72 are central regulators of ISR; both transcription factors EIN3 and MYB72 function as key regulators of microbe mediated ISR. Abbreviation—GSH: glutathione, ROS: reactive oxygen species, SAM: S-adenosyl methionine, ACC: 1-aminocyclopropane-1-carboxylic acid, KMBA: keto-4-methylthiobutyric acid, Na^+^: sodium ions, K^+^: potassium ions, HKT1: sodium transporter, SOSs: salt over sensitive, EIN3: ethylene insensitive, ERFs: ethylene response factors, HSFA2: heat shock factor A2, *APX2*: ascorbic peroxidase 2, *HSP18.2*: heat shock protein 18.2, ISR: induced systemic resistance, MYB72: R2R3 transcription factor.

The plant life cycle is associated with complex microbial communities, including fungi, bacteria, protists and viruses, all of which can impact various phases of plant growth, development and health. Plant-associated microbes directly alleviate biotic and abiotic stress using diverse mechanisms, for example, producing protective compounds, providing useful nutrients, degrading toxic compounds, etc. These microbes can also fine-tune plant hormone levels and the pathways that navigate plant growth. In the case of ethylene, numerous mechanisms that involve ethylene have been studied by which microbes can affect plant growth under abiotic stresses. Plant-microbial interactions can influence many regulatory steps of the ethylene biosynthesis pathway [4]. In this context, endophytic microbes can adjust ethylene concentration in plants through ACC deaminase activity, which converts plant ACC into ammonia and α-keto-butyrate. In this mechanism, microbes reduce plant ACC levels and protect from higher ethylene concentrations, which can be inhibitory to plant growth [87]. Most ACC deaminase-producing bacteria were isolates from the rhizosphere and were successful in protecting the plants against biotic and abiotic stresses [22,87,88,89,90]. Nowadays, the ACC deaminase enzyme is considered as one type of plant growth-promoting characteristic of endophytes. There is an ample amount of literature showing that ACC deaminase-producing endophytic bacteria of different genera such as *Bacillus*, *Pseudomonas*, *Streptomyces* and *Isoptericola* can promote plant growth under stress conditions [91]. Bacteria-inoculated plants showed lower levels of lipid peroxidation, ABA, and ethylene and higher levels of chlorophyll and IAA when compared to non-inoculated plants in drought stress [92,93,94,95,96]. Similarly, endophytic *Pseudomonas migulae* 8R6 and *Pseudomonas fluorescens* YsS6, with ACC-deaminase promoted the growth of tomato plants under salt stress [97]. To confirm the role of ACC-deaminase activity, the two endophytes were mutated in their ACC-deaminase activity. The results of inoculated plants showed that the mutant inoculated plants were less fit when compared to wild type-inoculated plants under salt stress. Similarly, rice seedlings inoculated with *Pseudomonas stutzeri* A1501 alleviated heavy metals and salt stress. All inoculated plants demonstrated higher fresh weight, longer roots and higher dry weight. The mutation of ACC deaminase compromised the plant growth promotion by the bacteria [98]. Therefore, these data indicate that bacterial ACC deaminase activity plays an important role in plant growth promotion under stress by reducing ethylene levels. However, the role of ethylene in mutualistic interactions is more complicated, as several reports have demonstrated its participation in different ways. Some plant-growth-promoting bacteria degrade the ethylene precursor ACC with an ACC deaminase, which presumably promotes the growth of the microbes by repressing the ethylene-induced host defense system [99]. There are also examples where ethylene shows a detrimental effect on microbial colonization. For instance, the external application of ethylene was shown to negatively influence nodulation in legume-rhizobia symbioses in *Medicago truncatula* [100]. Ethylene also restricts the growth of *Glomus aggregatum* arbuscular mycorrhizal (AM) fungus in pea (*P. sativum*) [101]. In addition, ethylene has been suggested as a negative regulator in the early phases of the symbiotic interaction of *M. truncatula* with the mycorrhizal fungi *Rhizophagus intraradices* and *Endogone versiformis* [24]. However, these responses are strain specific. For example, the exogenous application of ethylene negatively affects the virulence of *Agrobacterium*, leading to a decreased pathogenicity [102]. In contrast, some other bacteria such as, *P. aeruginosa*, *P. fluorescens, P. syringae* and *P. putida* respond positively to ethylene produced in plants [103]. The effect of ethylene on microbial plant colonization also involves cross-talk with other hormones. For instance, the cross-talk between two hormones, ABA and ethylene, regulates the establishment of AM symbiosis in tomato plants. ABA is necessary for arbuscule formation as ABA deficiency results in a low abundance of arbuscules in mycorrhizal roots. This low abundance is attributed to enhanced ethylene content due to ABA deficiency, which functions as a negative regulator of mycorrhizal colonization [104]. Similarly, the plant hormones and ethylene have interconnecting roles in mutualistic symbionts between *Laccaria bicolor* and *Populus* root interactions. The application of ACC and JA repressed the Hartig net formation, which is the secondary stage of colonization and alters the gene expression of cell wall biosynthesis and maintenance genes, suggesting that these two hormones reduce the Hartig net formation in roots. In addition, genes regulated by ethylene and jasmonic acid were regulated in the late stages of the interaction between *L. bicolor* and *Populus* [105]. Although all these studies underpin a deleterious effect of ethylene on root colonization by mycorrhizal fungi, it appears that plants need an optimum concentration of ethylene, as the lower concentration of ethylene is critical for plant growth and development, whereas the higher concentration of ethylene is usually known to inhibit plant growth [23]. Hence, the higher ethylene levels could be inhibitory for root colonization by AM, while lower concentrations might promote AM colonization. Therefore, the amount of synthesized ethylene is essential for AM establishment. These results also suggest that perhaps ethylene production activates the plant’s immune system which blocks the establishment of mutualistic symbioses. However, other studies show the opposite effects of ethylene in the interaction between plants and beneficial microbes [25]. For instance, the interaction of *Piriformospora indica* with barley and *Arabidopsis* confers salt stress tolerance; the fungus positively modulates the expression of ACC synthase and ethylene has been implicated as a positive modulator of the symbiosis, but no ACC deaminase has been found in the *P. indica* genome. The transcriptome data of barley roots colonized by *P. indica* showed that the fungus reprograms the major metabolic and transcriptomic processes including the ethylene biosynthesis pathway under salt stress. *P. indica* induces ethylene synthesis in *Arabidopsis* upon colonization and *Arabidopsis* mutants impaired in ethylene signaling show less colonization of the fungus. The fungus also enhances methionine synthase which might further induce ethylene biosynthesis [23,99]. These outcomes could be explained by the antagonism of ET and SA-mediated immune signaling. As mentioned before, SA based immunity is repressed by EIN3/EIL1 [86]. *P. indica* showed JA-dependent root colonization and suppression of SA-mediated immunity. Interestingly, EIN3/EIL1 are activated by the JA pathway and *P. indica* may recruit the ET and JA pathways to block SA-mediated immunity, which would otherwise effectively stop root colonization. Like *P. indica*, *Enterobacter* sp. SA187 alleviates salinity stress in *Arabidopsis*, which turns out to be 2-keto-4-methylthiobutyric acid (KMBA) dependent, plants converting KIMBA into ethylene. The transcriptome analysis of inoculated and non-inoculated plants revealed that, after SA187 inoculation, the expression of genes involved in photosynthesis and primary metabolism remains unchanged under salt stress conditions as compared to mock-inoculated plants (Figure 2). Under salt stress, for improved plant salt stress tolerance, both the beneficial microorganism and the host plant are required to have a coordinated regulation of the sulfur metabolic pathways. The ROS protection via glutathione (GSH) biosynthesis is tightly linked to sulfur metabolism and SA187 inoculated plants exhibit higher redox capacity by an enhanced ratio of GSH/GSSG (reduced/oxidized glutathione) compared to non-colonized plants under salt stress. In addition, glutathione also induces and regulates ethylene biosynthesis via regulating ACC-synthase. The exogenous application of GSH could recover stress tolerance in wild-type plants but not in ethylene insensitive2-1 mutant plants, showing that GSH-mediated resistance to salt stress happens via an ethylene-mediated pathway [106,107,108]. Similar to *P. indica*, SA187 also induces ethylene-responsive genes and inhibition of ethylene biosynthesis by AgNO_3_ resulted in a loss of the beneficial effects on plants under salt stress [107,108,109]. *Burkholderia phytofirmans* PsJN is one of the most explored PGPR, which is capable of promoting the growth of *Arabidopsis* plants [110,111]. In *Arabidopsis*, PsJN induces the growth of primary roots and root hairs, as well as aerial growth, increasing epidermal cell size [107] under salt stress [112]. *B. phytofirmans* PsJN did not induce plant growth in *ein2-1* mutants. The transcriptome showed up-regulation of *ACO* and *ACS* genes in root and aerial shoot parts, indicating a fine regulation of ethylene to induce salt tolerance in PsJN inoculated plants [112]. Recently, the role of ethylene has been shown in microbes-mediated plant adaptation to heat stress. Heat stress-induced ethylene signaling and heat stress transcription factors form a complex network of signal transduction that induces plant thermotolerance. In the context of beneficial microbes, the beneficial root endophyte, *Enterobacter* sp. SA187 induces thermotolerance in *Arabidopsis* by reprograming the plant transcriptome via ethylene. The root endophyte SA187 produces a sulfur-containing compound, 2-KMBA, which can be converted to ethylene by plants; in addition, plant ethylene signaling is also linked to the sulfur metabolism via SAM (S-adenosyl methionine) as a precursor of ethylene (Figure 2). This suggests that SA187produced compounds activate the ethylene signaling pathway in *Arabidopsis* which regulates plant thermotolerance by ERF-mediated higher expression of *HSFA2* and *HSPs* genes. Interestingly, SA187 primes the plants via EIN2- and HSFA2-dependent H3K4me3 modification of *APX2* and *HSP18.2* HS memory genes (Figure 2), thereby making plants more thermotolerant. This indicates that microbes induce constitutive plant thermotolerance via ethylene [62].

The modulation of ethylene due to microbial association with plants influences plant physiology and lifestyle. Research on PGPR has demonstrated their capability to control soil-borne pathogen attacks on plants in an ethylene-dependent manner. Beneficial microbes can induce systemic acquired resistance (ISR) in plants to suppress plant diseases (Figure 2). One such example is *P. fluorescens* WCS417r-mediated ISR against *P. syringae* in *Arabidopsis* and tomato. WCS417r induces *MYB72* and ethylene, which together result in ISR (Figure 2). The ethylene response mutant *etr1-1* lost the ability to be induced by rhizobacteria against *P. syringae*. Similarly, Arabidopsis plants treated with ACC showed enhanced resistance against *P. syringae* pv. tomato, indicating a role for ethylene in the microbes-mediated ISR in plants. Thus, responsiveness to ethylene seems to be essential for the induction of ISR; how ethylene regulates ISR is, however, not yet clear [113,114,115].

## 6. Conclusions

The higher expression of ethylene biosynthesis genes upon interaction with beneficial microbes indicates that ethylene signaling not only triggers against pathogenic microbes but also in response to beneficial endophytic microbes before recognizing them as a friend, perhaps to optimize the right amount of root colonization by the beneficial microorganisms. Ethylene as a gaseous hormone can also play a role in inter-plant communication, by warning adjacent plants about the coming danger in a plant community. The amount of ethylene present in plants affects the responses of their bacterial associates. Depending on the microbial strain, ethylene appears to be both a positive and negative regulator of mutualistic plant–microbial interactions. To understand the positive and negative role of ethylene on microbial colonization, further questions must be answered, such as whether ethylene impairs symbioses by affecting immunity, or whether cross-talk with other hormones are required. For example, ABA is necessary for arbuscule formation as ABA deficiency results in a low abundance of arbuscules due to enhanced ethylene content. In addition, understanding the role of ethylene in plant–microbe interactions probably requires broader experimental approaches using different ethylene concentrations, as well as investigating the interactions with other hormones in a crosstalk network. Plants can rapidly produce a large amount of ethylene as part of their physiological response to abiotic stresses. The increase in ethylene concentration prepares the plants to survive through environmental stresses, but can also negatively affect plant growth and lead to a decline in plant productivity, as higher concentrations of ethylene in plants may lead to plant senescence, chlorosis, and abscission. Thus, a broad screening of the ethylene concentration-dependent plant growth is needed to show to what extent plants respond to different ethylene concentrations. Such comprehensive information can help to answer several questions such as how various ethylene concentrations affect plant growth and how environmental, as well as internal signals, play a role in modifying ethylene responses in plants. In addition, it is important to compare gene expression patterns of plants treated with different concentrations of ethylene. Such data could help to understand the transcriptome responses that are specific to the positive and negative effects of ethylene on plant growth. Moreover, the understanding of the cell-types that are the main site for ethylene action in various abiotic stresses will contribute significantly to our knowledge of plant growth under normal and stress conditions. A complete understanding of ethylene-targeted transcription factors is critical for beneficial microbe-mediated plant growth promotion. So far, studies suggest that ethylene might have different effects on different stages of plant–microbe interaction to balance beneficial and non-beneficial characteristics of symbiosis. This is highly possible considering the huge amount of ethylene targets and ethylene-targeted transcription factors. There are over 100 ERFs that may affect positive and negative responses ranging from development and metabolic processes to defense gene activation. Therefore, ethylene is undoubtedly one of several important factors that play a role in mutualistic beneficial and negative non-beneficial interactions of plants and microbes.

## Data Availability

Not applicable.
